# Spatial Patterns of Genomic Variation and Genomic Offset in a Common Grassland Plant and Their Relation to Seed Transfer Zones

**DOI:** 10.1002/ece3.72152

**Published:** 2025-09-25

**Authors:** Johannes Höfner, Anna Bucharova, Walter Durka, Stefan G. Michalski

**Affiliations:** ^1^ Department of Community Ecology (BZF) Helmholtz Centre for Environmental Research ‐ UFZ Halle Germany; ^2^ Department of Biology University of Marburg Marburg Germany; ^3^ German Centre for Integrative Biodiversity Research (iDiv), Halle‐Jena‐Leipzig Leipzig Germany

**Keywords:** ecosystem restoration, genetic population differentiation, genomic offset, grasslands, seed sourcing, seed transfer zones

## Abstract

Restoring temperate grasslands often necessitates the introduction of large quantities of seeds, a process that is regulated by seed transfer zones in many countries. These zones are commonly delineated based on abiotic factors. Consequently, it remains uncertain to what extent existing seed zones represent and thereby protect or erode the spatial distribution of genetic variation. Empirical data on the spatial genetic structure of grassland species are therefore essential to address this knowledge gap. Moreover, as seed zones are increasingly expected to provide genotypes pre‐adapted to climate change, such data can also inform predictions of maladaptation and support the identification of suitable donor populations. Here, we focus on *Galium album* , a widespread perennial grassland species, which we sampled systematically across Germany, with an average of one population per 25 × 25 km area. Based on 8348 SNP loci, we analyzed the population genetic structure using Bayesian clustering. We identified four spatially coherent genetic clusters, which explained 2.43% of genomic variation but showed little congruence with current seed zones. Yet, seed zones still capture a significant component of spatial genetic structure (1.92%), which is also reflected in a significant isolation by distance among zones. Seed transfer practices are increasingly challenged by climate change, shifting the adaptive requirements for populations. We performed a genotype–environment association analysis using redundancy analysis, and estimated the genomic offset, that is, the genomic change required to maintain the current genotype‐environment relationship under climate change. The genomic offset was generally moderate across Germany, even under a pessimistic climate scenario projected into the more distant future (SSP5‐8.5, 2081‐2100). For one of the few locations where the temporal genomic offset slightly exceeded a previously proposed threshold, we identified suitable donor regions harbouring potentially pre‐adapted genotypes for targeted assisted migration, both within the same and in adjacent zones.

## Graphical abstract

Analysis of the genetic structure of *Galium album* , a widespread grassland species, across Germany revealed four spatially coherent genetic clusters that showed little congruence with current seed transfer zones, though zones still captured significant spatial genetic structure. Under climate change scenarios, genomic offset was generally moderate across Germany, suggesting current populations may be reasonably well adapted to future conditions. For locations where genomic offset exceeded recommended thresholds, suitable donor populations were identified in both the same seed zone and in adjacent seed zones that could provide pre‐adapted genotypes for assisted migration.

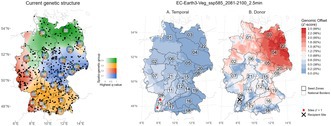

## 1 Introduction

Biodiversity is essential for the continued existence of Nature's Contributions to People on which we depend without alternatives (Díaz et al. 2019) and is considered a value in itself (e.g., White 2013). Grasslands are biodiversity record holders on spatial scales up to 50 m^2^ (Wilson et al. 2012) and cover 40% of the global land surface (Bardgett et al. 2021). Land‐use change threatens the extent of grasslands, 49% of which show signs of degradation (Gang et al. 2014). In Europe, for example, less than 25% of all grasslands are in a good state (European Environment Agency 2020). As conservation alone cannot revert these losses and maintain landscape multifunctionality (Aronson and Alexander 2013; United Nations 2015), it is vital to restore functional ecosystems (United Nations 2019; Tamburini et al. 2022).

In recent decades, grassland restoration requires increasingly large amounts of regional seeds. By definition, regional seeds evolve in the region of their application and are therefore adapted to the prevailing environmental conditions (Knapp and Rice 1994; Bucharova et al. 2017). Moreover, the use of regional seeds preserves existing spatial genetic differentiation. A common tool to regulate the sourcing, production and deployment of regional seeds is the use of seed transfer zones (‘seed zones’ hereafter). They are defined as ‘geographical regions within which plants can be moved with little or no consequences for population fitness’ (Hufford and Mazer 2003). When multiple sources from within a seed zone are mixed, a balance can be struck between maintaining current regional adaptation and increasing adaptability due to enhanced genetic diversity (Bucharova et al. 2019). Seed zones allow the development of a reliable and economically viable regional seeds production industry, scaling up restoration efforts (Zinnen et al. 2021).

The delineation of seed zones is challenging because of the general lack of species‐specific data on regional adaptation and genetic structure. In the absence of such data, seed zones based on ecoregions are commonly used (Ying and Yanchuk 2006; Bower et al. 2014; Cevallos et al. 2020). However, it is often not clear to which degree such provisional seed zones reflect the actual spatial genetic differentiation of plant populations. On one hand, genetic differentiation can be overlooked; on the other hand, provisional seed zones may be overly restrictive (Miller et al. 2011; Heenan et al. 2023). To validate the use of provisional seed zones or suggest improvements to them, it is thus essential to describe patterns of genetic differentiation (Hufford and Mazer 2003; Mijangos et al. 2015; Listl et al. 2018; Massatti et al. 2020; Rossetto et al. 2023).

Climate change constitutes yet another challenge for seed zones and the use of regional seeds. Local adaptation of populations is formed by past natural selection, and thus inherently reflects past conditions. To maintain adaptation in a changing climate, the allele frequencies within populations will have to shift either by natural selection from standing genetic variability or by gene flow from other populations. Intuitively, as the genetic shift required to maintain the same level of adaptation under climate change increases, the likelihood that a population will manage to adapt decreases (McKay et al. 2005). The magnitude of this genetic shift in time, required for local adaptation to keep pace with climate change, is called temporal genomic offset (Fitzpatrick and Keller 2015). The temporal genomic offset can be calculated using candidate adaptive loci and environmental data from the present and projected future (Capblancq et al. 2020; Gougherty et al. 2021). Where the temporal genomic offset is high, populations might have difficulties adapting in the future (Lachmuth, Capblancq, Prakash, et al. 2023). In the context of ecological restoration and seed sourcing, the method of genomic offset also enables the evaluation of the suitability of donor sites for a given recipient site.

When local standing genetic variation is not sufficient to adapt to changing conditions, suitable seed material can come from elsewhere, either by natural gene flow or by human‐mediated transfer. Mixing multiple sources of seed from within a seed zone, as is the case with *regional admixture provenancing*, could in some cases provide genotypes suitable for restoration under climate change (Bucharova et al. 2019). If the entire seed zone provides no such climate‐adjusted seeds, targeted assisted migration beyond the borders of seed zones might be needed (Lachmuth, Capblancq, Keller, and Fitzpatrick 2023), although this method is not without risks (Twardek et al. 2023; Rushing 2024, but see McKone and Hernández 2021).

**FIGURE 1:**
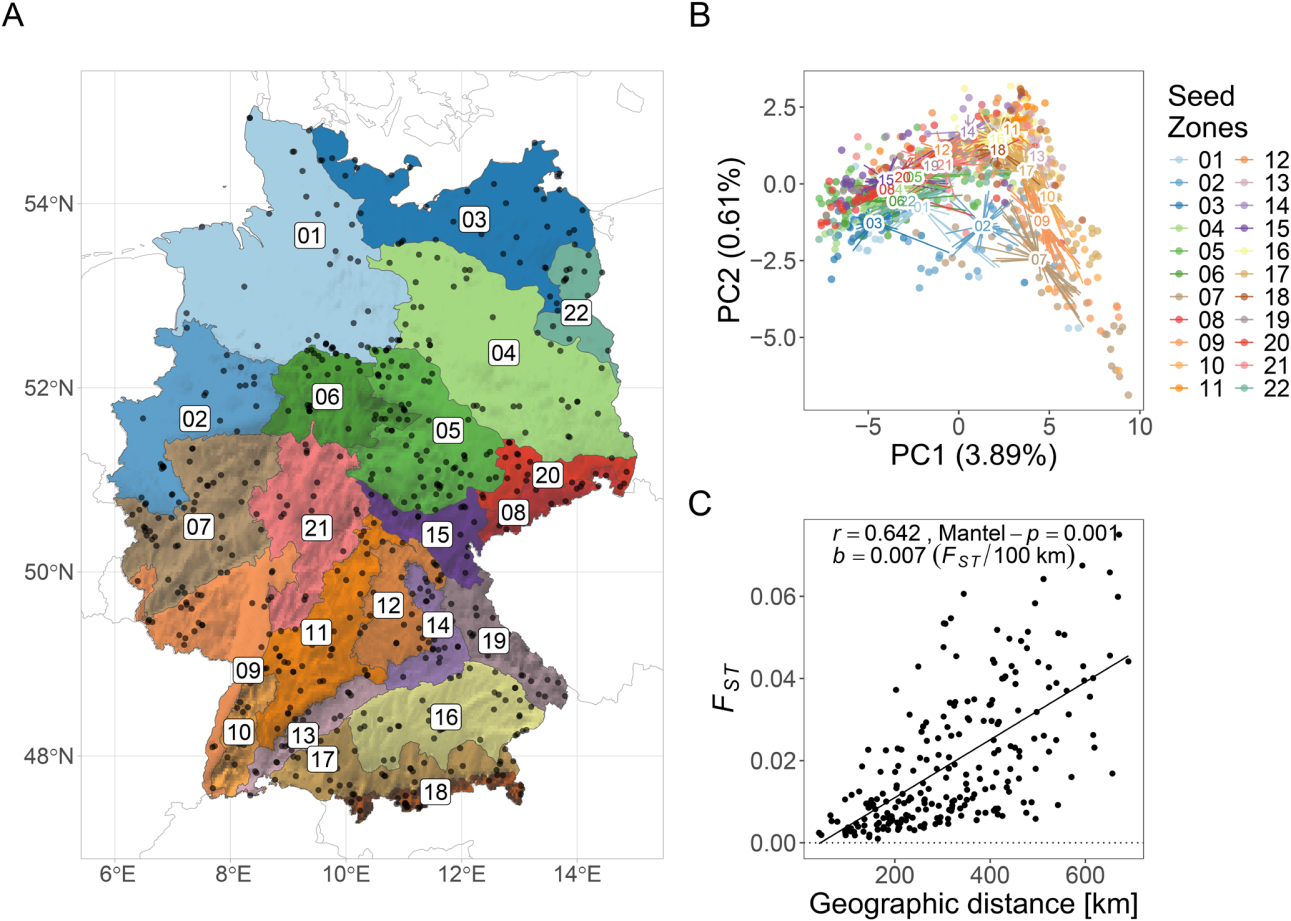
(A) German Seed zones (colours and numbers) and sampling sites (black dots). Shading represents elevation. (B) Principal Component Analysis (PCA) of the 735 individuals in the final data set. Colours analogous to panel A. Centroids marked by seed zone number. Coloured line segments point halfway from centroids of seed zones towards individuals. (C) Pairwise genetic differentiation between the 22 German seed zones (as *F*_ST_) as a function of geographical distance. *r*, Correlation coefficient; Mantel‐*p*, *p*‐value from Mantel test; *b*, slope of the linear regression.

The challenge posed to seed zones by climate change is especially evident when a legally binding system of seed zones for grassland restoration is already in place, such as in Germany (BNatschG 2009; ErMiV 2011). Its 22 zones (Figure 1A) apply to all common and widespread grassland plant species (Bucharova et al. 2019). The seed zones are largely based on previously described ecoregions (Meynen and Schmithüsen 1953–1962), which in turn are based on abiotic parameters such as geomorphology, geology and climate. However, it is unclear how well the seed zones capture the existing genetic variation of natural grassland populations and how suitable they are for climate change‐resilient restoration.

In this study, we used a comprehensive SNP dataset of samples covering all of Germany to describe the patterns of genetic variation of *Galium album* , a common grassland herb. We thereby built on previous findings based on a limited number of populations, where isolation by distance and relatively strong population differentiation compared to other species had been found (Durka et al. 2017). In addition, we expanded the approaches for evaluating genomic offset and donor importance described by Lachmuth, Capblancq, Prakash, et al. (2023). Based on these approaches, our study addresses four questions: (1) To what extent do geography, environmental conditions and demographic history shape the spatial genetic structure of *G. album* across Germany? (2) How do the observed spatial genomic patterns relate to the seed zones? (3) How much genomic turnover is necessary for populations to adapt to future climates? (4) Where are suitable donor populations for those locations where the temporal genomic offset is high?

## 2 Materials and Methods

### 2.1 Study Taxon

*Galium album* Mill. (Rubiaceae) is a tetraploid, perennial herb and is considered to have originated from diploid *G. mollugo* s.str. (Krendl 1967; Natali et al. 1995). While both species are widely distributed across Europe, *G. album* is much more common in Germany (Fagerlind 1937; Jäger 2016). *G. album* is self‐incompatible and obligately outcrossing (Crowe 1964), and the flowers are predominantly pollinated by Syrphidae, Muscidae and Larvivoridae (Ančev and Krendl 2011). Its seeds lack dedicated dispersing structures. The species typically grows in pastures and mesic grassland.

### 2.2 Study Design and SNP Data Set

**FIGURE A1:**
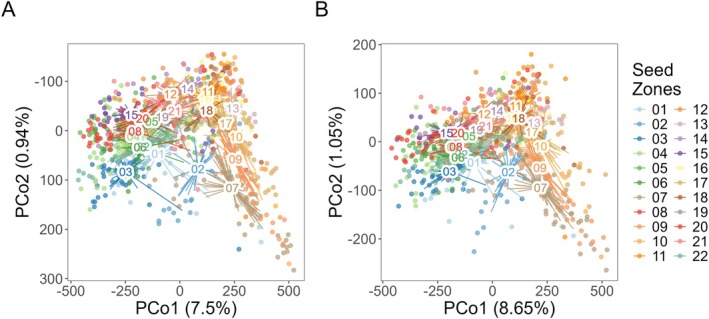
Principal Coordinates Analysis (PCoA) of *G. album* individuals (A) with diploid genotyping as used in all downstream analyses and (B) based on allele frequencies from raw reads. The y‐axis of (A) has been inverted to match (B) and the main PCA (Figure 1B). The Φ_ST_ values from the corresponding AMOVA are 0.056 and 0.060 for diploid genotyping and allelic dosage, respectively. The similarity of these values and of (A) and (B) illustrates that proceeding with diploid genotyping for methodological compatibility is justified.

Here, we used the data set of *Galium album* from the project *RegioDiv*, where volunteers had collected 985 leaf samples from all across Germany (for detailed methods, see Durka et al. 2025). For genotyping, we used the double digest restriction site‐associated DNA sequencing (ddRAD) protocol by Peterson et al. (2012) with minor modifications. Libraries were sequenced and co‐dominant, biallelic, single‐nucleotide polymorphism (SNP) markers were derived using dDocent (Puritz et al. 2014; O'Leary et al. 2018). We filtered to a minimum allele frequency of 0.05 and retained a single SNP per contig. Since a comparative analysis with allelic frequencies based on raw allele counts showed highly similar patterns of genetic structure and differentiation among seed zones (Figure A1), we applied diploid genotyping to allow for consistent and comparable analyses across taxa in the *RegioDiv* project (Durka et al. 2025). Lastly, we cleaned the data set (1) by removing individuals that had been collected as *G. album* by mistake, using taxonomic reference samples, and (2) by excluding loci that were responsible for batch effects using OutFLANK (Whitlock and Lotterhos 2015). The resulting data set consisted of 735 individuals originating from 534 sites, genotyped at 8348 loci. We identified putative cpDNA loci by a BLAST search of the reference sequences obtained from dDocent against the nt database of GenBank (accessed 31 March 2021), using the programme blastn (‐db nt\‐task megablast\‐evalue 1.0e‐6). If not otherwise stated, all following analyses were performed in R 4.3.1 (R Core Team 2023). We identified clusters of individuals based on putative cpDNA loci only using the ‘find.clusters’ function from the ‘adegenet’ package (Jombart 2008).

### 2.3 Environmental and Climate Data

To characterise present and future environmental site conditions, we used climatic data from WorldClim2 (Fick and Hijmans 2017) and soil data from SoilGrids 2.0 (Poggio et al. 2021). We chose an Earth model that considers vegetation development (EC‐Earth3‐Veg‐LR, Smith et al. 2014). We assumed constant soil conditions for the present and future. Accordingly, for the present (1970–2000) and the future (2081–2100), we combined the 19 bioclimatic and all 11 soil variables from SoilGrids250m 2.0 into a common grid with 2.5′ resolution. We excluded *bio08* (mean temperature of wettest quarter) and *bio09* (mean temperature of driest quarter) from further analysis, as they feature abrupt, biologically meaningless changes in the landscape for the present (1970–2000) in Germany. This exclusion is especially justified given that these variables are correlated with other variables (e.g., bio15 and bio19). To explore the effect of climate change, we first modelled the most extreme case under the worst available emission scenario (SSP5‐8.5) and the timeframe farthest into the future (2081–2100). As the genomic offset was moderate even in this rather extreme setting (see Section 3.2), we did not explore less pessimistic scenarios, as their impact can be expected to be even less severe.

**FIGURE A2:**
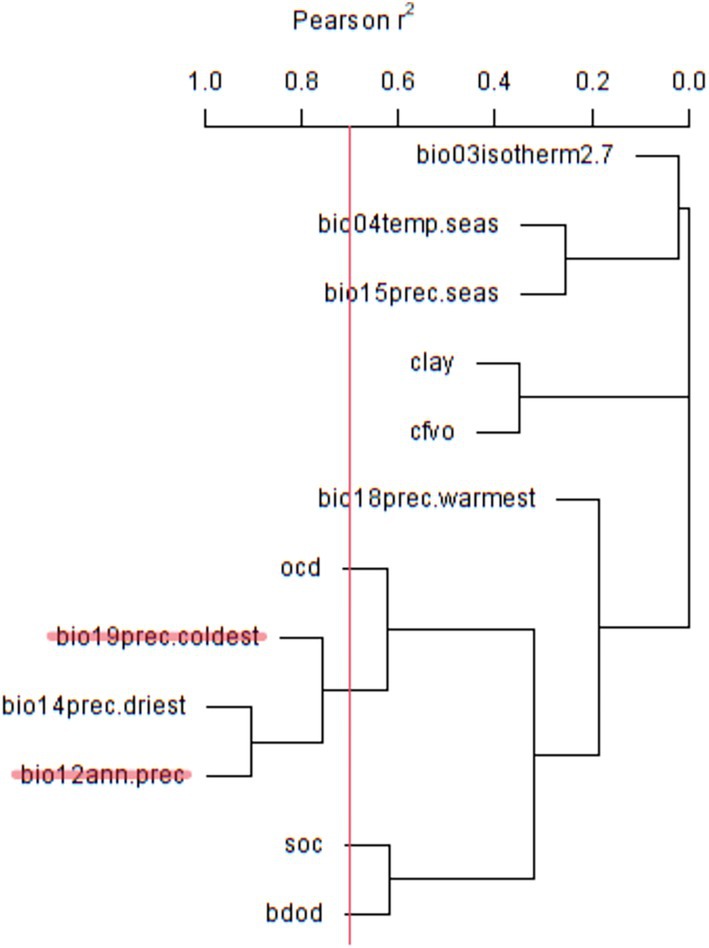
Correlation dendrogram of variables identified by the forward model selection approach based on the Spearman correlation coefficient. The red line indicates the cutoff at *ρ* ^2^ = 0.7, used to keep only uncorrelated variables. According to the cutoff, only one branch has redundancies (bio19, bio14 and bio12). Of that branch, we kept bio14 as it was identified first by the forward selection procedure. Red strikethrough designates discarded variables.

To identify independent environmental variables associated with genetic structure, we used the forward model selection approach *sensu* Blanchet et al. (2008). A model of genetic variance was built by iteratively adding environmental variables to the model, maximising the explained genetic variance with the ‘ordiR2step’ function from the ‘vegan’ package (Oksanen 2022). The stopping criteria were *p* ≤ 0.01 (based on 1000 permutations), a decrease in adjusted *R* ^2^, or surpassing the adjusted *R* ^2^ of a full RDA with all environmental variables as explanatory variables. The forward model selection process identified 12 environmental variables. We clustered the identified variables according to the Spearman correlation coefficient using the ‘varclus’ function from the ‘Hmisc’ package (Harrell 2023), resulting in eight branches below a Spearman correlation coefficient of 0.7 (Figure A2). We selected the most plausible variable per branch for further analyses (Table 1).

**TABLE 1:** Independent bioclimatic and soil variables from WorldClim2 and SoilGrids 2.0, selected for their association with genetic variation.

Variable name	Description
isotherm2.7	BIO3: Isothermality (mean diurnal temperature range divided by annual temperature range)
prec.driest	BIO17: Precipitation of the driest month
prec.seas	BIO15: Precipitation seasonality
prec.warmest	BIO18: Precipitation of the warmest quarter
temp.seas	BIO4: Temperature seasonality
bdod	Bulk density of the oven‐dry fine earth fraction
cfvo	Volumetric content of fragments larger than 2 mm in the whole soil
clay	Soil clay content in the fine earth fraction (%, 0–5 m)
ocd	Organic carbon density
soc	Soil organic carbon content in the fine earth fraction (g/kg, 0–5 cm)

### 2.4 Genetic Population Structure Analysis

If not stated otherwise, all genetic population structure analyses have been performed on the full set of loci including those identified as cpDNA. First, we visualised genetic relationships of individuals via principal component analysis (function ‘glPCA’, package ‘adegenet’; Jombart 2008). We assessed population structure using the model‐based Bayesian clustering algorithm of Admixture 1.3.0 (Alexander et al. 2009), which, given a number of ancestral populations, assigns individuals to them (‘genetic clusters’), allowing for admixture. We varied the prescribed number of ancestral populations in Admixture's model (*K*) from one to 22. We based our decision for the most plausible (‘optimal’) number of clusters (*K*_opt_) on both Admixture's cross validation output (‐cv flag) and biological plausibility: With increasing number of *K*, we regard *K* as biologically plausible when the newly added cluster is mostly geographically contiguous and contains any individuals fully assigned to it (i.e., cluster membership *q* > 0.9). We acknowledge that genetic structure is often hierarchical and complex. There might be no ‘true’ number of ancestral populations. We spatially interpolated Admixture's *Q*‐matrix across Germany using the ‘Krig’ function from the ‘fields’ package (Nychka et al. 2021). For visualisation as a map we colour‐coded the clusters, and visualised the highest *q*‐value per grid cell (hereafter referred to as ‘spatio‐genetic groups’).

Overall genetic differentiation among seed zones was estimated via an analysis of molecular variance (AMOVA) using the ‘poppr’ package (Kamvar et al. 2014) with the ‘ade4’ method (Dray and Dufour 2007). For comparison, a second AMOVA was conducted in which individuals were assigned to *K*_opt_ spatio‐genetic groups according to spatial interpolation described above. Overall *F*_ST_ of seed zones was calculated using the ‘basic.stats’ function from the ‘hierfstat’ package (Goudet 2005). Pairwise genetic differentiation between seed zones was estimated as *F*_ST_ values applying the ‘stamppFst’ function from the ‘stampp’ package (Pembleton et al. 2013). In order to test for isolation by distance (IBD), we correlated the matrix of pairwise *F*_ST_ values with a matrix of geodesic distances between mean coordinates of the sites in a given seed zone, testing for significance with a Mantel test. We used partial redundancy analysis (pRDA) to estimate the independent relative influence of geography, demographic history and environment on allelic frequencies. For geography, we used the plain geographic coordinates of the sampling sites; for demographic history, the ancestry coefficients from Admixture at *K*_opt_; and for environment, the selected environmental variables (Table 1) at the sampling sites. Since RDA is sensitive to missing values, we imputed missing genotypes using the ‘impute’ function from the ‘LEA’ package (Frichot and François 2015) with the number of ancestral populations equal to *K*_opt_. We then calculated a full model including geography, environment and demographic history as explanatory variables to obtain the total explained variance (Table 2). To isolate the effects of the explanatory variables, we calculated models for each variable separately, each with the remaining variables as co‐variables.

**TABLE 2:** Variance partitioning using pRDA. G: allele frequencies of the full set of loci; geo: coordinates of the sampling site; demo: demographic history given as Admixture's *Q*‐matrix at *K* = 4; env: environmental variables. Significant *p* values with *α* = 0.05 are shown in bold.

Model	Formulae	Prop. of explained variance	Prop. of total variance	*p*
Full model	G ~ env + geo + str	1	0.071	**0.001**
Pure environment	G ~ env | (geo + str)	0.328	0.023	**0.001**
Pure demographic history	G ~ str | (env + geo)	0.102	0.007	0.477
Pure geography	G ~ geo | (env + str)	0.205	0.015	**0.001**
Confounded		0.365	0.026	

### 2.5 Adaptive Landscape and Genomic Offset

**FIGURE A3:**
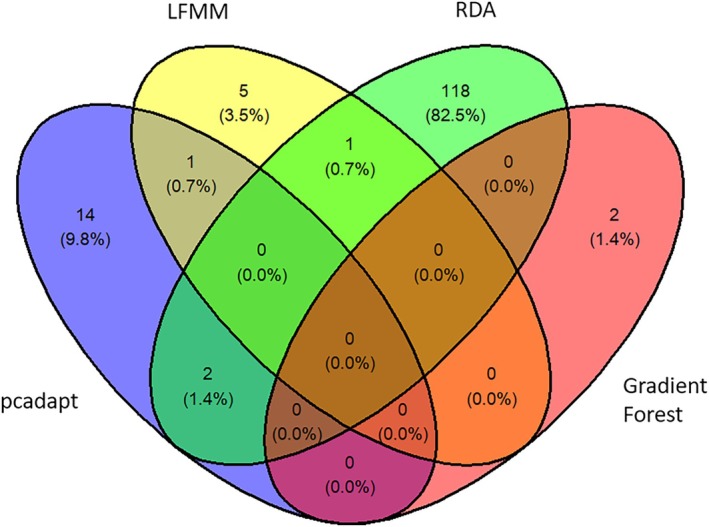
Venn diagram of the candidate adaptive loci identified by four different methods: pcadapt, LFMM, RDA and Gradient Forest. We used the four loci identified by more than one method for the calculation of the ‘adaptively enriched’ RDA (Capblancq and Forester 2021).

For the calculation of adaptive indices and genomic offsets, we largely followed Capblancq and Forester (2021). To identify candidate adaptive loci based on genotype–environment association, we used four methods: pcadapt, LFMM, RDA and Gradient Forest (Figure A3). First, we used pcadapt (Luu et al. 2017) to identify candidate adaptive loci by detecting SNPs that show unusually strong correlation with population structure. This population structure was inferred from the first two principal components of overall genetic variation. Second, we applied latent factor mixed models (LFMMs) based on an exact least‐squares approach to identify loci showing significant correlations with environmental gradients, while accounting for population genetic structure using the LEA package (Frichot and François 2015). The environmental gradients were described by the bioclim variables and the soil variables as selected above. We then ran the ‘lfmm2’ function with the number of latent factors equal to *K*_opt_. The *p* values for the association between loci and environmental variables were obtained using the ‘lfmm2.test’ function for the full set of environmental variables. To correct for false discovery rate, only loci with significant *q*‐values (*α* < 0.05) were retained (Benjamini and Hochberg 1995). Third, we used redundancy analysis (RDA) to model SNP allelic frequencies by environmental predictors, while accounting for demographic history. The function ‘rdadapt’ then identifies outliers in environmental space based on Mahalanobis distances (Capblancq et al. 2018), incorporates an inflation factor (François et al. 2016) and calculates *q*‐values. We retained loci with *q* < 0.05. Lastly, for the application of Gradient Forest (Ellis, Smith and Pitcher 2012), we removed major population structure effects from the SNP data by calculating residuals from a linear regression of SNP allelic frequency on the first two principal components of overall genetic variation. We fit the Gradient Forest model with these residuals as a response and the same environmental variables as above as predictors, using 1000 bootstrapped trees. As an ad hoc threshold, we then considered the top 5% of loci most strongly associated with the environment as putative adaptive loci.

**FIGURE A4:**
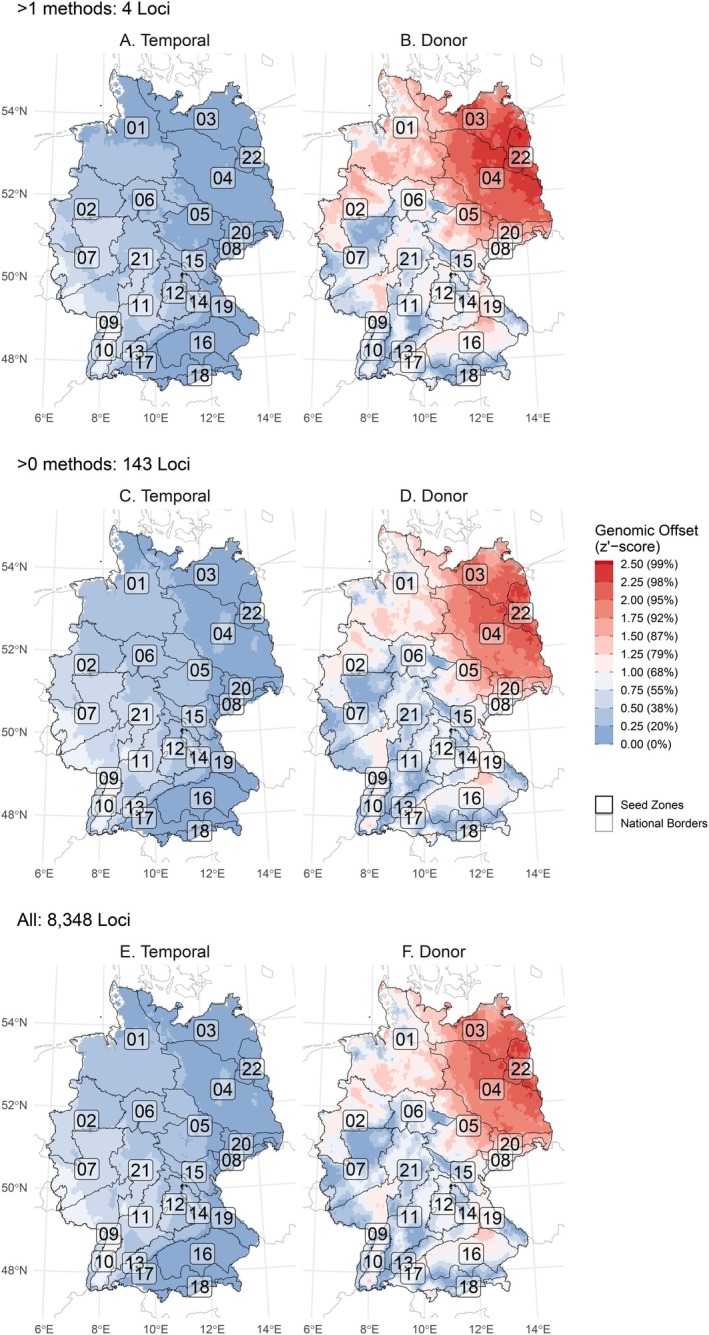
Comparison of the patterns of genomic offset resulting from different sets of candidate adaptive loci: Loci identified by more than one of the four methods (pcadapt, LFMM, RDA and Gradient Forest), loci identified by at least one method, and all loci. Due to the similarity of the results, we used the most conservative set of the four loci that were identified by more than one method.

We evaluated the effect of the choice of candidate adaptive loci on downstream patterns of genomic offset by comparing the results of using (1) the four loci identified by more than one method, (2) the 143 loci identified by any method, and (3) all loci (Figure A4). As the differences were negligible, we opted for the conservative approach to keep only the four loci identified by more than one method. Indeed, genome‐wide loci with low diversity may predict genotypes at candidate adaptive loci (Bertin et al. 2020) and genome‐wide variation may be more relevant to conservation than any putatively adaptive variation we may identify (Kardos et al. 2021; Bruxaux et al. 2024, but see e.g., Dauphin et al. 2020).

**FIGURE A5:**
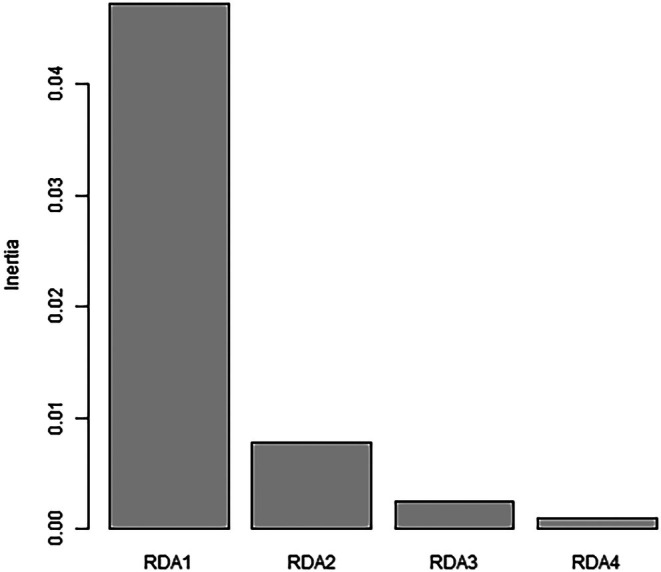
Scree plot of eigenvalues of the adaptively enriched RDA.

We calculated an adaptively enriched RDA, by describing allele frequencies of candidate adaptive loci per sampling site with environmental variables (Capblancq and Forester 2021). Using this RDA, an adaptive index can be calculated. Adaptive indices are per‐RDA‐axis values that sum up the environmental values of a given grid cell, weighted by the association of the environmental variables with the respective RDA axis. Calculated for all grid cells across our study area, the resulting ‘adaptive landscape’ shows a linear combination of the environmental variables that is relevant to the loci associated with that axis. The adaptive landscape is interpreted as genomic turnover due to changing environmental conditions. In a procedure similar to Steane et al. (2014), the adaptive index is calculated as ${\text{Adaptive Index}}_{r,c}=\sum_{i=1}^{n}a_{i}\;b_{i}$ with *r*, RDA axis; *c*, grid cell; *i*, environmental variable; *a*, the variable's loading; *b*, standardised value of variable. Following Capblancq and Forester (2021), we calculated the adaptive index for the first two RDA axes, which captured most of the variance explained by the adaptively enriched RDA (Figure A5). Using projected environmental conditions, we calculated adaptive indices for the future as well.

Genomic offsets are distances in the adaptively enriched environmental space *sensu* Steane et al. (2014). Therefore, given this space, a genomic offset can be calculated between any two points in space and time for which environmental data is available. Lachmuth, Capblancq, Prakash, et al. (2023) distinguish spatial offsets between different locations in the same time period, temporal (local) offsets within one location between different time periods (Fitzpatrick and Keller 2015), and spatio‐temporal offsets across space and time. We calculated the temporal offset for each grid cell between its present (1970–2000) and future (2081–2100) conditions using the ‘genomic_offset’ function from Capblancq and Forester (2021) with the first two RDA axes. These temporal genomic offsets represent how large the climate change‐induced disruption of genotype‐environment associations is expected to be for a given grid cell. We transformed each temporal genomic offset into a *z*′‐score by adapting the standardisation procedure of the offsetEnsembleR package (https://github.com/SusanneLachmuth/offsetEnsembleR)—originally formulated for Gradient Forest outputs—to our RDA‐based offsets, thereby situating each cell's future‐present offset within the empirical distribution of all contemporary spatial offsets in the study area (Germany). This reference distribution of all contemporary, spatial genomic offsets represents the present‐day environmental variation relevant to the adaptation of *G. album* populations. The *z*′‐scores follow the Empirical Rule (Ross 2017), that is, a *z*′‐score of 1 corresponds to the 68th percentile of the reference distribution of all spatial contemporary genomic offsets, a *z*′‐score of 2 corresponds to the 95th percentile. However, the *z′*‐scores have no meaning in terms of standard deviations of the reference distribution (hence *z*′ instead of *z* like in Lachmuth, Capblancq, Prakash, et al. 2023).

In the absence of common garden data, *z* = 1 has been suggested as a reasonable not‐to‐exceed threshold above which plant performance is likely to decrease substantially (Lachmuth, Capblancq, Keller, and Fitzpatrick 2023). Accordingly, we used *z*′ = 1 as a threshold to identify areas in Germany where the genotype‐environment association of *G. album* is expected to be severely disrupted by climate change. For a selected site for which adaptive disruption is predicted, we calculated a spatio‐temporal ‘donor offset’ to identify suitable climate‐adjusted donor sites. This donor offset is equivalent to the entries in the scaled offset matrix from Lachmuth, Capblancq, Keller, and Fitzpatrick (2023). Accordingly, we defined suitable donor sites as those with a donor offset of *z*′ < 1 between their current conditions and the projected future conditions of the recipient site.

## 3 Results

### 3.1 Genetic Population Structure

A total of 4.76 × 10^9^ sequence reads, that is, on average 4.83 × 10^6^ reads per sample, were used for SNP detection and genotyping, resulting in 4.28 × 10^6^ raw SNPs. After filtering, 8348 biallelic SNPs of 735 samples originating from 534 sites remained, with 16.6% of missing data.

**FIGURE A6:**
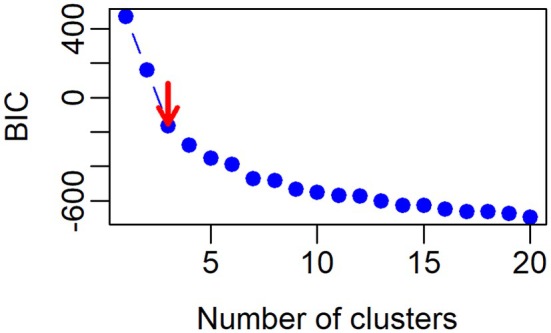
Bayesian Information Criterion (BIC) output from adegenet::find.clusters() run on individuals with the putative cpDNA loci only.

**FIGURE A7:**
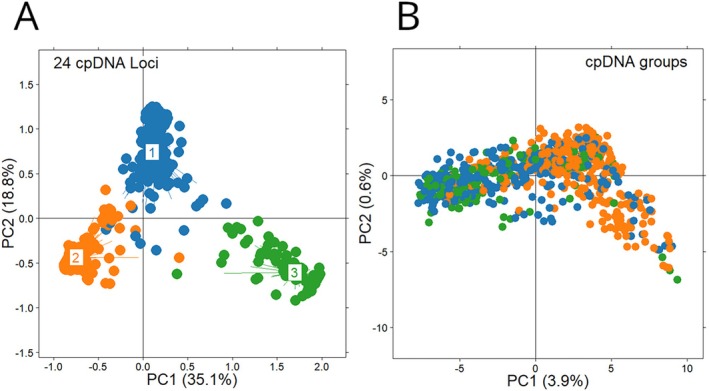
Principal Component Analysis (PCA) of all individuals (A) using only the 24 loci identified as cpDNA, and (B) using all loci and individuals (cf. Figure A12A). The cpDNA haplogroups identified by k‐means from panel (A) are colour‐coded.

**FIGURE A8:**
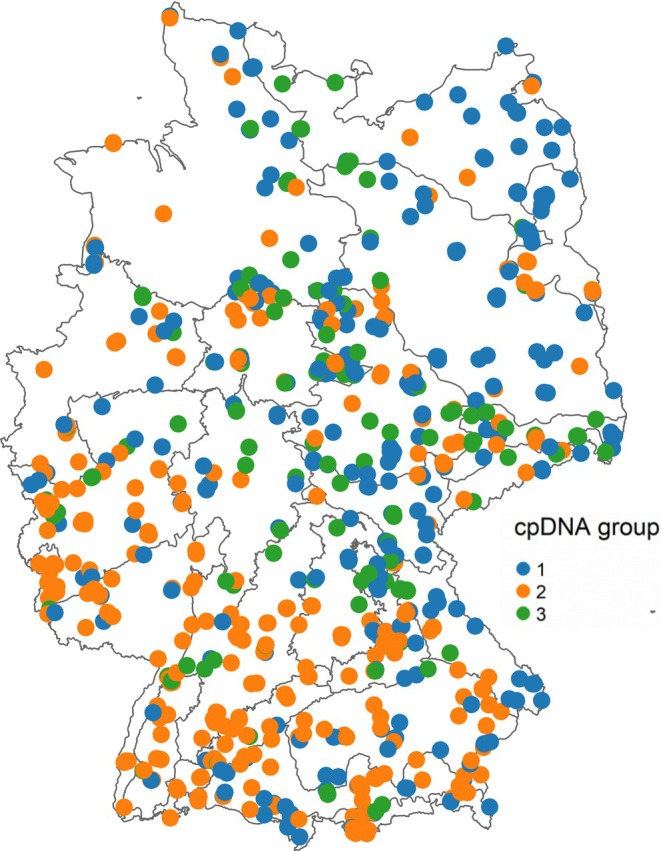
Distribution of the identified cpDNA haplogroups (*N* = 3) in Germany.

Out of 8348 loci, we identified 24 as putatively belonging to cpDNA. Individuals clustered into three distinct cpDNA groups (Figures A6 and A7). All groups were present across Germany, with Group 1 (blue) predominating in the northeast, Group 2 (yellow) predominating in the southwest and Group 3 (green) being mostly restricted to Central Germany (Figure A8). All following results stem from the full set of 8348 loci including those identified as cpDNA.

**FIGURE 2:**
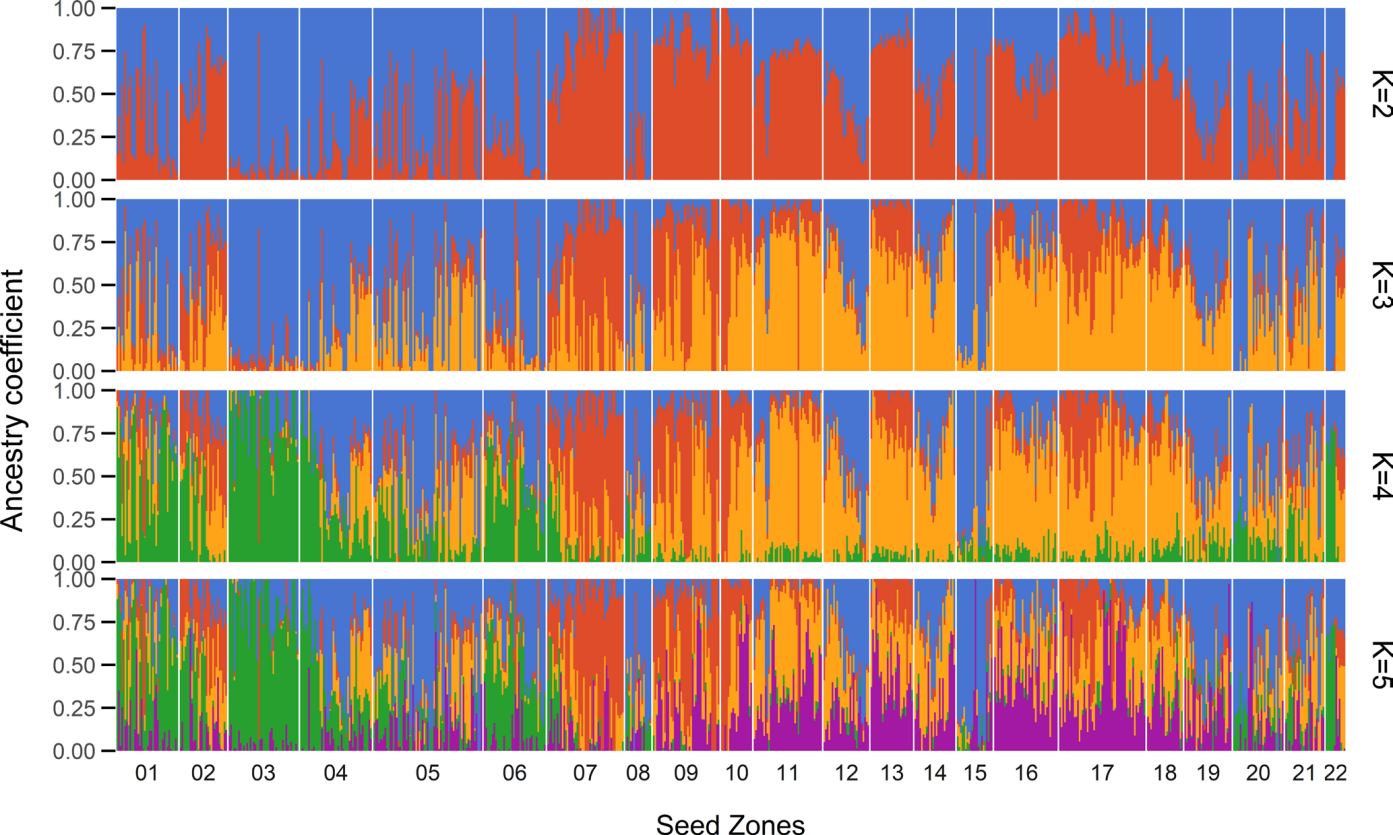
Ancestry coefficient plot of the Admixture results. One vertical bar represents one individual, its colours the partial assignments to ancestral populations. Each row of bars corresponds to one predefined number of ancestral populations. Horizontal gaps between bars separate seed zones.

**FIGURE 3:**
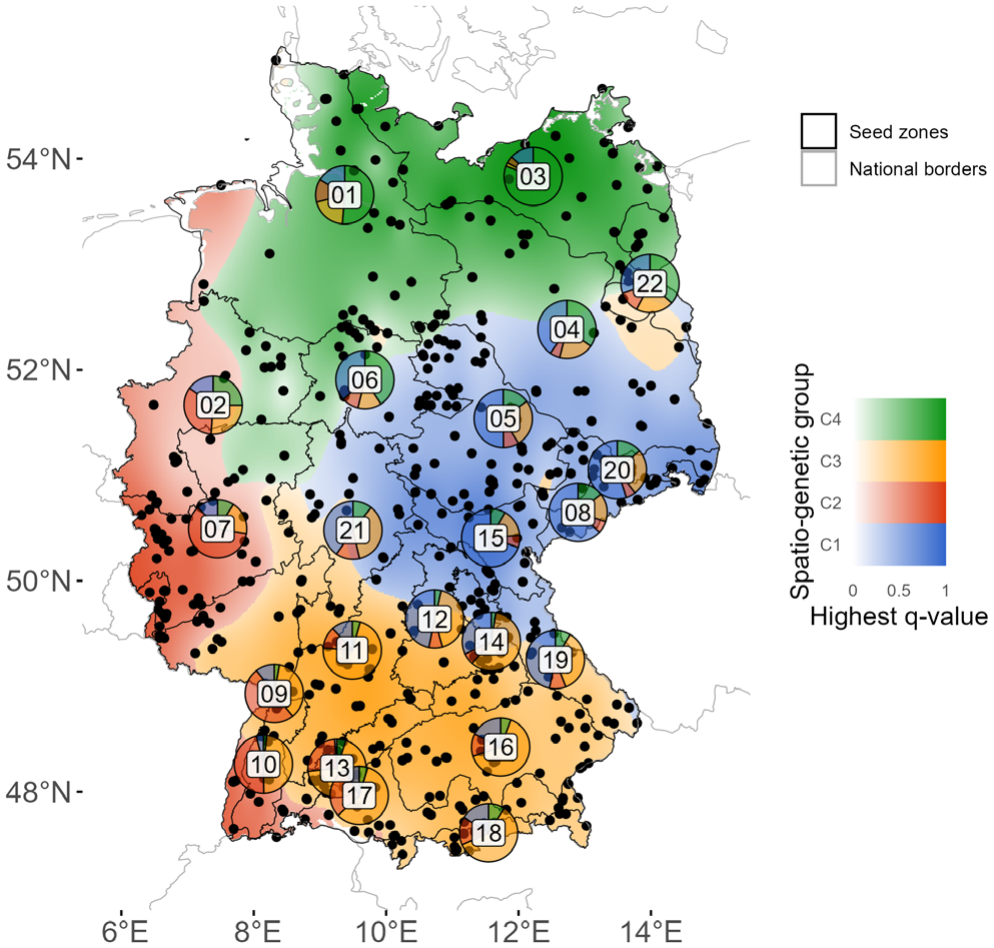
Spatio‐genetic groups (spatial interpolation of the Admixture results at *K* = 4). Colour fills indicate spatio‐genetic groups. Transparency shows the highest *q*‐value. Numbered pie charts indicate mean cluster memberships across all individuals per seed zone.

**FIGURE A9:**
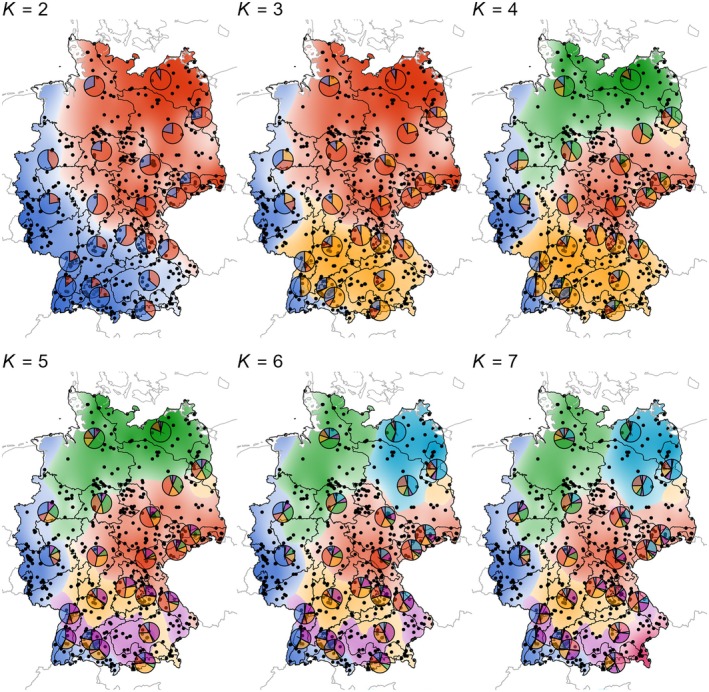
Spatial interpolations of the Admixture results with the number of ancestral populations varied from *K* = 2 to *K* = 7. The colours of the clusters and spatio‐genetic groups correspond to those in Figure 3.

**FIGURE A10:**
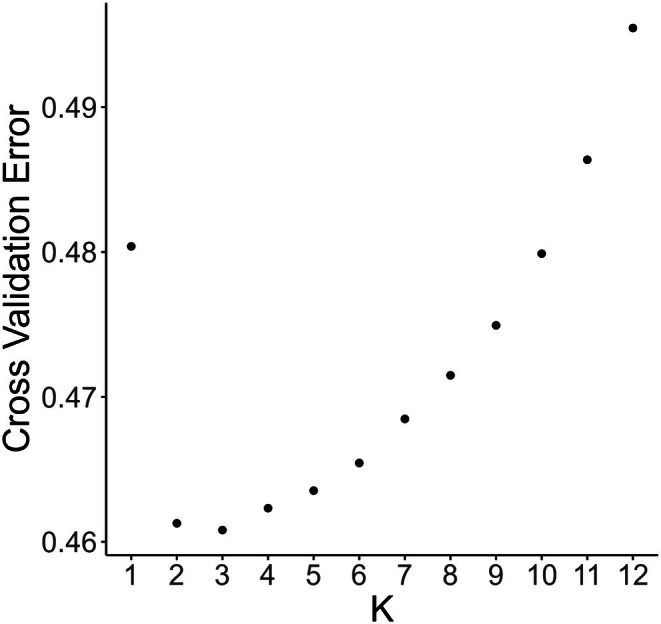
Cross‐entropy plot to determine the statistically optimal number of ancestral populations for the Admixture analysis.

The clustering results of the Admixture analysis at *K* = 2 distinguished the north‐east of Germany from the southwest (Figure 2, Figure A9). Many individuals were fully assigned (*q* > 0.9) to either genetic cluster, and the zones were often dominated by one cluster (e.g., zone 03 dominated by blue, zone 07 dominated by red). However, mixture and admixture were found in other zones (e.g., zones 01, 04, 05). The statistically optimal solution as per cross‐entropy analysis was *K* = 3 (Figure A10). At this level, the westernmost parts of zones 07, 09 and 10 were separated from the initial south‐western cluster. At *K* = 4, the initial north‐eastern blue cluster was subdivided latitudinally into a northern (green) and a southern (blue) part, with the transition cutting through zones 06, 04 and 22 (Figure 3). At *K* = 5, the newly defined cluster lacked sufficient non‐admixed individuals (Figure 2), and substantial spatial incoherences of the clusters emerged (Figure A9). Therefore, we regarded four clusters (north, south, west and central) as the most plausible solution (*K*_opt_ = 4). In this solution, the clusters are mostly spatially coherent in northern, central and southern Germany, and the fourth cluster predominates in a narrow band along the western border, suggesting potential genetic continuity with populations in adjacent countries. The corresponding spatio‐genetic groups rarely concur with the seed zones (Figure 3). More than half of the zones encompassed multiple spatio‐genetic groups, indicating within‐zone genetic heterogeneity (Figure 3).

Partial redundancy analysis (pRDA, Table 2) attributed genetic variation to geographic location (coordinates), the selected environmental variables (five bioclimatic variables and five soil variables), or demographic history (the ancestry coefficients at *K*_opt_ = 4, Table 2). The full model including all three variable sets explained 7% of all genetic variation (‘explained variance’ hereafter). Environment alone accounted for most of the genetic variance (33%), corresponding to 2% of the total variance. Geography alone accounted for 21% of explained variance, and demographic history alone explained the least with 10% of explained variance. The remaining 36% of explained variance was confounded between the three sets of variables.

**FIGURE A11:**
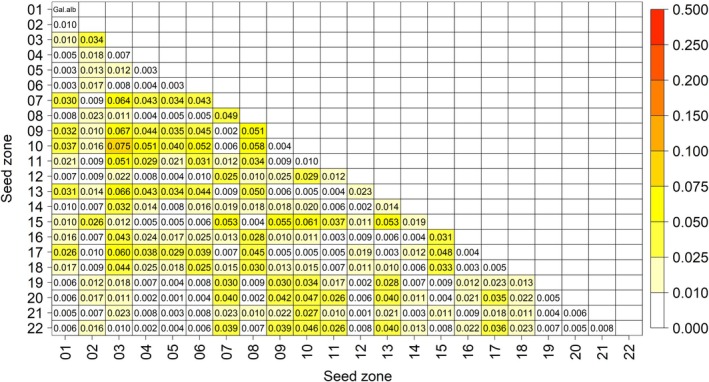
Matrix of genetic differentiation (as *F*_ST_) between the German seed zones. The differentiation values are colour‐coded as indicated by the colour bar on the right. All values were significant (*p* < 0.05).

**FIGURE A12:**
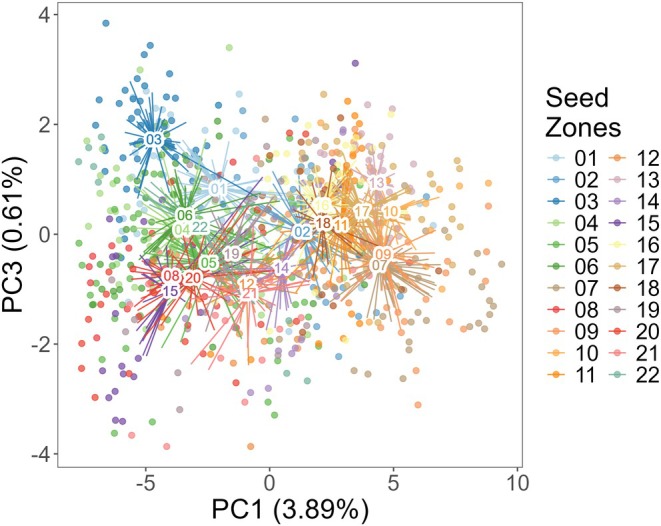
Principal Component Analysis (PCA) of all individuals using all loci, showing PC1 and PC3.

The seed zones were genetically differentiated (Figure A11). Principal component analysis (PCA) corroborated this finding, since the seed zone centroids were clearly separated in the ordination (Figure 1B, Figure A12). Individual samples across the zones had large genetic overlap. Some zones were more genetically heterogeneous (e.g., 09, 21) than others (e.g., 03, 13), as indicated by differences in sample spread. The first axis corresponded to a west–east gradient, while the second axis differentiated northern from southern populations. A western group including zones 07 and 09 stood slightly apart from the main cluster. Significant genetic differentiation was also detected by AMOVA, where the zones accounted for 1.92% of the total variance (Φ_ST_ = 0.019, Table A1). Spatio‐genetic groups reflecting the coloured cluster areas in Figure 3 explained 2.43% of genetic variance. The global *F*_ST_‐value among zones was 0.0096 and all 253 pairwise *F*_ST_‐values were significant (Figure A11). We also found a significant pattern of isolation by distance between the zones, with a slope of 0.007 *F*_ST_/100 km and 41.2% of explained variance (Figure 1C).

### 3.2 Adaptive Index and Genomic Offset

**FIGURE A13:**
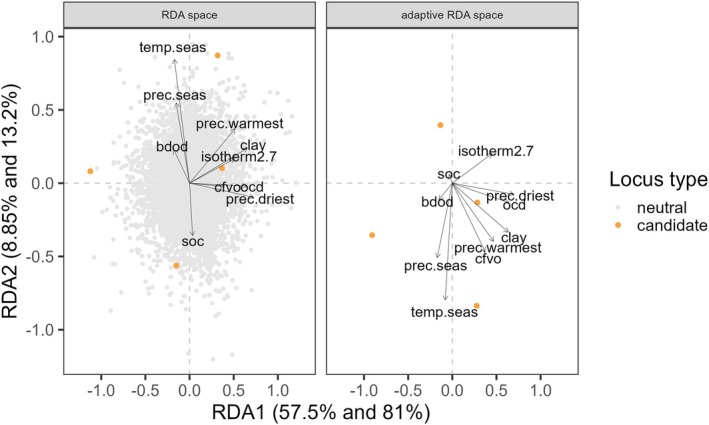
Biplots of Redundancy Analysis (RDA) showing loci and the selected environmental variables along the first two axes. Percentages on the axes represent their proportion of explained variance. (A) RDA used as one of the four methods to identify candidate adaptive loci. In this RDA, all genotypes are the response matrix, and the selected environmental variables are the explanatory matrix, with the Admixture ancestry coefficients at *K*_opt_ = 4 (‘demographic history’) as conditioning variables (G ~ env|demo). Loci scores rescaled ×20 for readability. The orange dots represent the loci that have been identified as candidate adaptive loci by more than one method. (B) Adaptively enriched RDA with the adaptive loci as the response matrix and the selected environmental variables as the explanatory matrix (G.adapt ~ env). This RDA is the basis for adaptive indices and genomic offsets.

**FIGURE A14:**
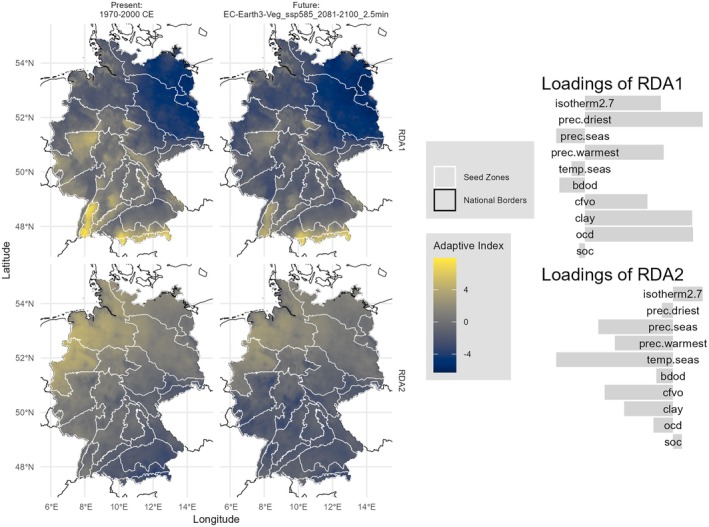
The adaptive index of the first two RDA axes across the study area (adaptive landscape, unweighted by eigenvalues), calculated with environmental values of the present (1970–2000) and the projected future (2081–2100, EC‐Earth3‐Veg, SSP5‐8.5). The adaptive index can be understood as the value on an axis in an environmental space that is warped according to its association with candidate adaptive loci (‘adaptively enriched space’, Capblancq and Forester 2021). Horizontal bars on the right: Loadings of the axes with the selected environmental variables with bars to the left showing the magnitude of negative loadings and bars to the right showing positive loadings.

We used four methods for finding genotype‐environment associations, which together identified 147 loci, 143 of which were identified by one method only. Pcadapt identified 17 loci, LFMM seven, RDA 121 and Gradient Forest two loci (Figure A3). We considered the four loci that were identified by more than one method as candidate adaptive loci (see Section 2.5). The adaptively enriched RDA was calculated with the allele frequencies at the candidate adaptive loci as response variables and the environmental variables previously selected as explanatory variables. The first axis of the adaptively enriched RDA was positively associated with the precipitation of the driest month, the precipitation of the warmest quarter of the year, isothermality, soil clay content and soil organic carbon density (Table A2, Figure A13). Accordingly, lower adaptive indices were found for the drier and sandier northeast of Germany, while higher values were found for mountain ranges (Figure A14). The adaptive indices of the first axis for the projected future suggested that populations of *G. album* will need to adapt to drier warm seasons by the end of the century. The second axis of the adaptively enriched RDA mainly represented the seasonality of temperature and precipitation. Correspondingly, the adaptive landscape of the second axis showed a continentality gradient that increases to the southeast. For the projected future, it suggested mostly a requirement to adapt to reduced seasonality and potentially an overall wetter summer.

**FIGURE 4:**
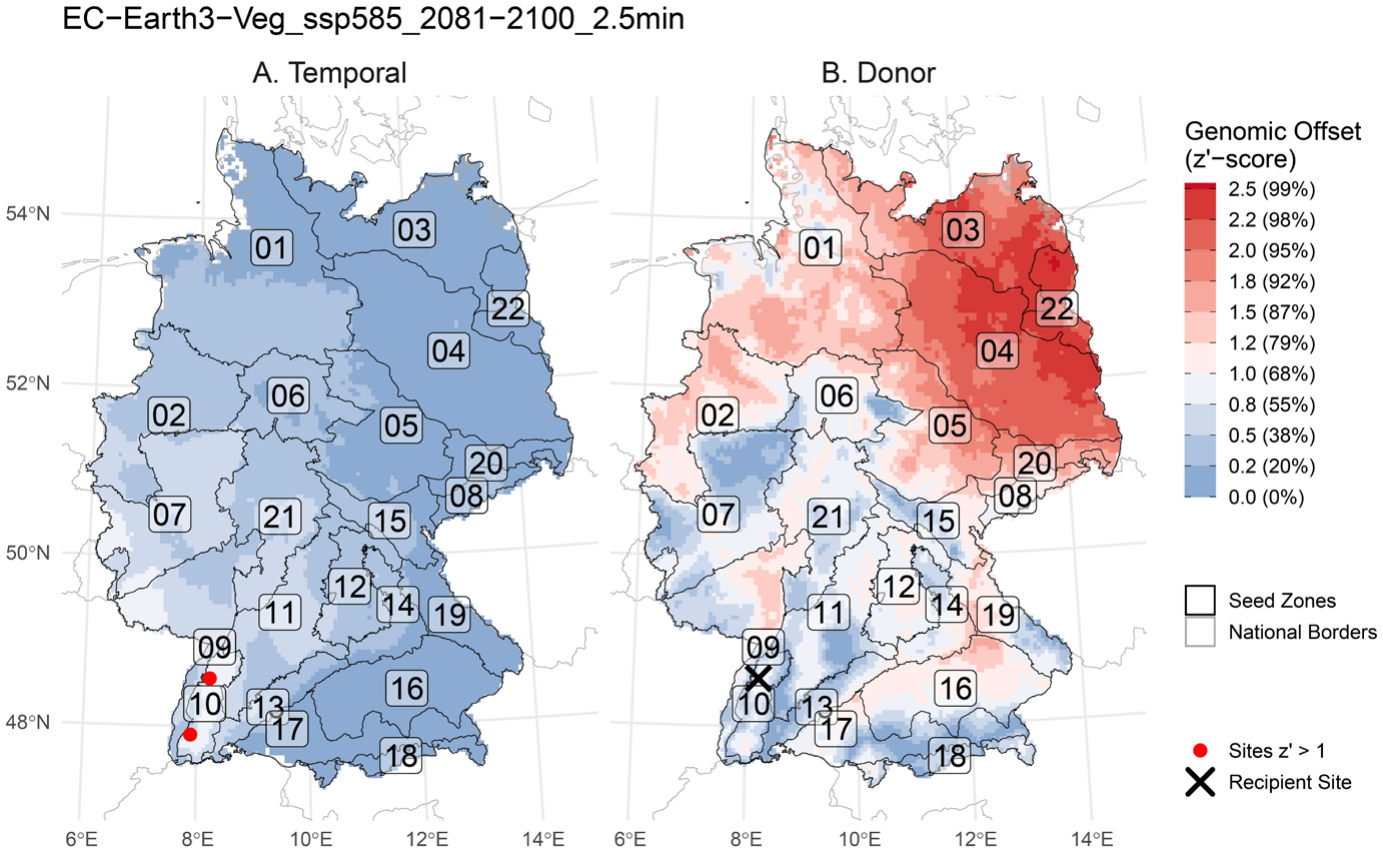
Spatial distributions of different genomic offsets. The offsets are re‐expressed sensu Lachmuth, Capblancq, Prakash, et al. (2023). The resulting *z′*‐score still follows the Empirical Rule (e.g., *z*′ = 1 corresponds to the 68th percentile of all spatial contemporary offsets, see percentages in brackets), but has in this case lost its meaning in terms of standard deviations. Blue represents ‘acceptable’ *z′*‐scores lower than the not‐to‐exceed threshold of 1, red represents ‘unacceptable’ *z′*‐scores greater than the not‐to‐exceed threshold of 1. (A) Temporal genomic offset between the present (1970–2000) and the future (2081–2100 with model EC‐Earth3‐Veg and scenario SSP5‐8.5). There were two grid cells where the threshold was slightly exceeded (red dots). (B) Donor offset for an exemplary recipient site (black diagonal cross). Populations from blue areas are considered suitable donor sites for the given future projection. The darker the blue, the more suitable the donor population.

When converted to *z′*‐scores, the temporal genomic offsets ranged from < 0.01 to 1.01 (Figure 4A), with a tendency to increase towards the southwest. They were highest in and around the Western German uplands in seed zones 07, 09, 10, 11 and 21. Accordingly, populations of *G. album* in these regions are expected to experience higher disruption in genotype‐environment associations (GEA) under projected future conditions, potentially leading to excess maladaptation in the future that may surpass the populations' adaptive potential. Two grid cells (< 1‰ of all cells, see black arrows in Figure 4A) in zone 10 (Black Forest) exceeded the ad hoc not‐to‐exceed threshold of *z'* = 1 and were therefore formally considered vulnerable to projected future conditions. We randomly chose one of these cells as an exemplary seed recipient cell and calculated the future climate‐adjusted donor suitability of all cells (Figure 4B). Most of zone 10 itself was a suitable donor area, as were mountain ranges of the southwest of Germany. Most low‐elevation areas, especially the dry and sandy northeast, were unsuitable donors.

## 4 Discussion

We used a data set of SNP loci to infer geographic genetic structure in *Galium album* , a common plant of European grasslands, within Germany. We found significant isolation by distance and four biologically plausible and spatially coherent genetic clusters, potentially representing ancestral phylogeographic groups. According to partial redundancy analysis, genomic variation was mostly attributed to a combination of the predictors, followed by environmental conditions and geography alone, and to a lesser extent to demographic history. The genetic structure only partially aligns with the legally binding grassland seed zones in Germany. We identified four candidate adaptive loci potentially conveying environmental adaptation, and used them to estimate maladaptation of the populations under projected future conditions. Under a relatively pessimistic climate scenario, we found that the genomic offset remained below the ad hoc not‐to‐exceed threshold across nearly the entire study area. For one location predicted to experience maladaptation, we used the genomic offset to identify suitable climate‐adjusted donor areas.

### 4.1 Genetic Structure

The structure of total genetic diversity in *Galium album* is shaped mostly by environmental factors and geography, and to a lesser extent by demographic history. These factors explained 33%, 21% and 10% of the explained variance, respectively (Table 2). A substantial part of the explained genetic variance (36%) was confounded among these factors, which is common for this type of data (Legendre 1993; Sork et al. 2016; Marková et al. 2023).

Environmental conditions (climate and soil) were the strongest individual predictor for genetic variance, accounting for 33% of the explained variance and 2.3% of the total genetic variance. Since this variance is uniquely attributable to environmental factors, unconfounded by demographic history or geographic distance, it likely reflects adaptive processes. Other studies have found higher proportions of total genetic variance explained exclusively by environmental conditions (Sork et al. 2016; Capblancq and Forester 2021; Chen et al. 2023), sometimes by an order of magnitude (Temunović et al. 2020; Jiang et al. 2025). However, direct comparisons between studies have to be examined with caution: differences in organismal life history (e.g., woody species vs. herbaceous), the number and selection of environmental variables, geographic scale, sampling density, and the number of retained SNPs all influence the proportion of variance attributable exclusively to environmental predictors.

Variation at adaptive candidate loci specifically was most strongly associated with precipitation, isothermality and several soil variables. The adaptive landscapes of the present reflect this pattern, suggesting adaptation to a warmer, drier climate and sandier soil in the north‐east and wetter, less continental conditions in the west, especially in the mountain ranges (RDA1 in Figure A14). According to the second axis of the adaptive landscape, populations may be adapted to greater temperature seasonality in the increasingly continental southeast. It should be noted, however, that the second axis explained substantially less genetic variance than the first.

Geography, represented by spatial distances, was another notable predictor for total genetic variance, accounting for 21% of the explained variance and 1.5% of the total genetic variance (Table 2)—a higher proportion than in most of the studies mentioned above (Sork et al. 2016; Temunović et al. 2020; Capblancq and Forester 2021; Chen et al. 2023; Jiang et al. 2025). Exclusive explanatory power of geography is consistent with the expectation that gene flow declines with increasing spatial distance, producing a pattern of isolation by distance (Hutchison and Templeton 1999). Indeed, we observed significant IBD in *Galium album* (Figure 1C), corroborating earlier findings based on AFLP markers and a more limited population set (Durka et al. 2017). Nevertheless, disentangling the specific contribution of restricted gene flow to genetic variation remains challenging, as geographic distance is inherently confounded with both demographic history and environmental heterogeneity (Orsini et al. 2013). In *G. album* , limited gene flow is plausible given that it relies on insect pollination and barochory.

Demographic history was the weakest individual predictor, accounting for 10% of explained variance. We hypothesise there were at least two distinct ancestral lineages of *G. album* that may have colonised Central Europe from east to west. The spatial differentiation between these hypothetical lineages (*K* = 2 in Figure A9) is supported by the cpDNA group distribution, where haplotype Group 2 predominates in the southwest, while Groups 1 and 3 occur primarily in the northeast and Central Germany, respectively (Figure A8), consistent with major postglacial migration routes. Similar patterns of postglacial recolonisation from both the west and the east were documented in other species, resulting in a longitudinal suture zone in Central Europe (Taberlet et al. 1998; Hewitt 1999).

We observed admixture in the ancestry coefficients, that is, individuals that were largely or entirely assigned to a genetic cluster that was not predominant in their region (e.g., red in zones 01, 06 and 08, blue in zone 11, Figure 2). Correspondingly, some individuals belonged to a cpDNA haplotype group uncommon in their region, sometimes occurring several hundred kilometres from the core range of the haplotype group (Figure A8). A likely explanation for this is human‐mediated dispersal: *G. album* is a species of seminatural, agriculturally used meadows and seeds could have been carried over long distances by livestock or agricultural machinery (Fischer et al. 1996). Moreover, *G. album* has been sown as part of seed mixtures for grassland restoration, and non‐regional material may have spread into the seminatural sites that we sampled (e.g., Gemeinholzer et al. 2020).

### 4.2 Seed Zones

When considered as populations, seed zones were significantly genetically differentiated with a global *F*_ST_ of 0.018 and explained 1.9% of the total observed genetic variance according to AMOVA (Table A1). This value is comparable to other SNP‐based studies on grassland plant species: For example, Michalski et al. (2017) found a global *F*_ST_ of 0.090 in *Arrhenatherum elatius* among eight locations from Italy to Sweden, and Conrady et al. (2022) found pairwise *F*_ST_ values from 0.016 to 0.143 (average 0.056) between regions in five grassland species. These *F*_ST_ values are much smaller than values reported in older studies that used markers with loci selected for their differentiating capability, like AFLP or microsatellites: A previous study on *G. album* in the same region using AFLP markers detected 14.8% of genetic variability explained by the region (Durka et al. 2017). Indeed, explained genetic variance is highly dependent on the marker type used (Ai et al. 2014). The genetic differentiation that we found between the seed zones is thus not negligible.

Although the seed zones (Figure 1A) are genetically differentiated, they only partially reflect the spatio‐genetic groups we identified (Figure 3), and seed zone borders rarely align with the borders of the spatio‐genetic groups. At *K* = 4, more than half of the zones encompass more than one spatio‐genetic group. For instance, the north–south elongated western spatio‐genetic group was spread across western parts of six seed zones (red at *K* = 4, Figure 3), while no zone was fully dominated by it. All in all, a substantial part of within‐species genetic variation in *G. album* is not covered by the current system of seed zones. This is common when seed zones are based on environmental proxies instead of genetic data (Massatti et al. 2020, however, see Miller et al. 2011), as are most seed zones (e.g., Bower et al. 2014; Cevallos et al. 2020; Rivière et al. 2022). The current system therefore bears the danger of homogenising a part of the existing genetic variance. Nonetheless, our IBD and RDA analyses suggest that genetic differentiation correlates with geographic distance and environmental distance. Consequently, seed zones defined by environmental criteria still capture a part of genetic differentiation, particularly at larger spatial scales. They reflect regional adaptation as well, at least to a certain extent (Kramer et al. 2015; Bucharova et al. 2017). Thus, ecoregions are still useful proxies for genetic variation in the absence of genetic data and when more detailed seed zones are impractical.

The four spatio‐genetic groups (Figure 3) explained substantially more genetic variation than the 22 seed zones (Table A1). Consequently, one could argue that it would be sufficient to have four seed zones for *G. album* . However, these zones would then span several hundred kilometres of a diverse adaptive landscape (Figure A14). In a species with significant isolation by distance, seed transfer based on a few large zones may homogenise and distort genetic differences within zones. This could be more problematic than using the current, smaller seed zones. Additionally, the seed zones in Germany are generalised, that is, they apply to all grassland plant species, which requires a compromise across species, sacrificing parts of species‐specific differentiation (St. Clair and Johnson 2004; Prasse et al. 2010). Therefore, smaller seed zones such as those in place are more likely to capture within‐species diversity across multiple species.

### 4.3 Adaptive Requirements for the Future

With ongoing climate change, existing adaptation of some plant populations lags behind the rapidly changing environmental conditions (Wilczek et al. 2014; Anderson and Wadgymar 2020). We used the temporal genomic offset to identify areas where populations of *G. album* will potentially experience elevated disruption of genotype‐environment associations in the future. We adapted the standardisation of genomic offsets introduced by Lachmuth, Capblancq, Prakash, et al. (2023) and Lachmuth, Capblancq, Keller, and Fitzpatrick (2023) to RDA‐based genomic offset (Capblancq and Forester 2021). Surprisingly, only a negligible part (0.007%) of the study area exceeded the ad hoc threshold of *z'* = 1 in their temporal genomic offset, even under the most pessimistic scenario. Above *z'* = 1, it is increasingly unlikely that adaptation will keep pace with the change in environmental conditions (Lachmuth, Capblancq, Prakash, et al. 2023), in our case with regard to the period 2081–2100. Areas where *G. album* populations exceeded the *z'* = 1 threshold were rare and small (Figure 4A), which may reflect that a grassland plant species as common and widespread as *G. album* has substantial genetic variation, conferring sufficient adaptive potential for even drastic environmental change. While the dry season might become drier in Central Europe, other seasons may become wetter (IPCC 2021), potentially benefiting some species (Doležal et al. 2022). Moreover, land‐use change rather than climate change is responsible for most grassland degradation in Europe (Liu et al. 2019). While most populations of *G. album* across Germany are predicted to face no major adaptive disruptions, isolated exceedances such as in zone 10 (Black Forest) suggest that even an otherwise resilient species like *G. album* may face localised maladaptation risks.

As an example of practical relevance, we identified suitable donor areas for one location in zone 10 (Black Forest) which exceeds the temporal genomic offset threshold of *z′* = 1. We used the entries in the scaled offset matrix from Lachmuth, Capblancq, Keller, and Fitzpatrick (2023) and Lachmuth, Capblancq, Prakash, et al. (2023) as a ‘donor offset’, demonstrating its utility to identify candidate donor populations for a given recipient site. In the present climate, zone 10 occupies one extreme of the adaptively enriched environmental space, characterised by milder dry seasons and low seasonality (Figure A14). In the future, this shifts towards more severe dry periods and more seasonality, which negatively impacts grasslands (Fischer et al. 2020). These conditions can be found in other parts of zone 10 today, as well as in a few other low‐mountain ranges outside of zone 10 in the west of Germany, making them suitable donors (blue in Figure 4B). This suggests that the only climate‐vulnerable populations that we identified can receive climate‐adjusted material from within the same zone if the right donor sites are chosen.

Genomic offset analysis offers a means of assessing the potential vulnerability of populations to climate change. The present analysis provides an essential first step toward assessing the effectiveness of the current German seed zones for restoration under climate change. Future research should widen the scope of the analysis across multiple species relevant to restoration. This would enable identification of common patterns and a broader assessment of generalised seed zones. However, genomic offset analyses should not serve as the sole basis for management decisions. One limitation is that the genomic offset‐based donor suitability does not account for any genomic variation beyond the selected loci. As a result, in the presence of isolation by distance, out of two locations with equal donor suitability, the one closer to the recipient site should be preferred. Moreover, independent validation of these results is needed (Rellstab et al. 2021), for instance through common gardens and climate manipulation experiments. Common gardens would allow the definition of a case‐specific empirical *z*‐score threshold, beyond which population performance declines too much (Lachmuth, Capblancq, Keller, and Fitzpatrick 2023). When these limitations are addressed, the genomic offset can help identify areas vulnerable to environmental change and provide valuable guidance for climate‐adjusted seed transfer in restoration.

## Author Contributions

**Johannes Höfner:** conceptualization (equal), formal analysis (lead), investigation (supporting), methodology (equal), writing – original draft (lead), writing – review and editing (equal). **Anna Bucharova:** conceptualization (supporting), investigation (supporting), writing – review and editing (equal). **Walter Durka:** conceptualization (equal), data curation (lead), formal analysis (supporting), investigation (equal), methodology (equal), project administration (lead), writing – review and editing (equal). **Stefan G. Michalski:** conceptualization (equal), formal analysis (equal), investigation (equal), methodology (lead), project administration (supporting), writing – review and editing (equal).

## Conflicts of Interest

The authors declare no conflicts of interest.

## Data Availability

Demultiplexed raw sequence data are available in the European Nucleotide Archive (ENA) at EMBL‐EBI under accession numbers PRJEB71395 (https://www.ebi.ac.uk/ena/browser/view/PRJEB71395) and PRJEB94855 (https://www.ebi.ac.uk/ena/browser/view/PRJEB94855). Accession numbers of samples used and a genotype‐containing R object are accessible under the following doi: https://doi.org/10.5281/zenodo.17094223.

## Appendix A

**TABLE A1:** Results from two AMOVAs, one based on current seed zones and one based on the spatio‐genetic groups (Figure 3, see Section 2). AMOVA have been calculated using the R‐package ‘poppr’ (Kamvar et al. 2014) with the ‘ade4’ method (Dray and Dufour 2007). In AMOVA, statistics are *F*‐statistic analogues calculated in an ANOVA‐like procedure with pairwise haplotype distances (Excoffier et al. 1992). Accordingly, higher values of Φ indicate higher differentiation between populations. The Φ values correspond to Φ_ST_ (population‐total), Φ_IS_ (individual‐population) and Φ_IT_ (individual‐total), respectively.

	df	Sigma	%	Φ	*p*
Between seed zones	21	19.7617	1.9	**0.019**	0.001
Within seed zones	713	27.5088	2.7	0.027	0.001
Within samples	735	983.3779	95.4	0.046	0.001
Total	1469	1030.6484	100		
Between spatio‐genetic groups	3	25.2158	2.4	**0.024**	0.001
Within spatio‐genetic groups	731	28.281	2.7	0.028	0.001
Within samples	735	983.3779	94.8	0.052	0.001
Total	1469	1036.8746	100		

**TABLE A2:** Loadings of the first three axes of the adaptively enriched redundancy analysis (RDA) used for the genomic offset.

	RDA1	RDA2	RDA3
prec.driest	0.72	−0.09	0.17
soc	0.03	−0.35	0.42
temp.seas	−0.19	0.85	−0.19
ocd	0.64	−0.01	0.38
bdod	−0.18	0.22	−0.42
clay	0.65	0.24	−0.49
cfvo	0.38	−0.01	−0.46
prec.seas	−0.17	0.56	0.41
prec.warmest	0.51	0.38	0.55
isotherm2.7	0.52	0.16	−0.01
